# Mizagliflozin, a selective SGLT1 inhibitor, improves vascular cognitive impairment in a mouse model of small vessel disease

**DOI:** 10.1002/prp2.869

**Published:** 2021-09-29

**Authors:** Nanae Ishida, Maki Saito, Sachiko Sato, Yu Tezuka, Atsushi Sanbe, Eiichi Taira, Masamichi Hirose

**Affiliations:** ^1^ Division of Molecular and Cellular Pharmacology Department of Pathophysiology and Pharmacology Iwate Medical University School of Pharmaceutical Sciences Iwate Japan; ^2^ Department of Pharmacy Iryo Sosei University Fukushima Japan; ^3^ Department of Pharmacology Iwate Medical University School of Medicine Iwate Japan; ^4^ Division of Pharmacotherapeutics Department of Pathophysiology and Pharmacology Iwate Medical University School of Pharmaceutical Sciences Iwate Japan

**Keywords:** cerebral small vessel disease, mizagliflozin, SGLT1, vascular cognitive impairment

## Abstract

Previously, we showed that sodium/glucose cotransporter 1 (SGLT1) participates in vascular cognitive impairment in small vessel disease. We hypothesized that SGLT1 inhibitors can improve the small vessel disease induced‐vascular cognitive impairment. We examined the effects of mizagliflozin, a selective SGLT1 inhibitor, and phlorizin, a non‐selective SGLT inhibitor, on vascular cognitive impairment in a mouse model of small vessel disease. Small vessel disease was created using a mouse model of asymmetric common carotid artery surgery (ACAS). Two and/or 4 weeks after ACAS, all experiments were performed. Cerebral blood flow (CBF) was decreased in ACAS compared with sham‐operated mice. Phlorizin but not mizagliflozin reversed the decreased CBF of ACAS mice. Both mizagliflozin and phlorizin reversed the ACAS‐induced decrease in the latency to fall in a wire hang test of ACAS mice. Moreover, they reversed the ACAS‐induced longer escape latencies in the Morris water maze test of ACAS mice. ACAS increased SGLT1 and proinflammatory cytokine gene expressions in mouse brains and phlorizin but not mizagliflozin normalized all gene expressions in ACAS mice. Hematoxylin/eosin staining demonstrated that they inhibited pyknotic cell death in the ACAS mouse hippocampus. In PC12HS cells, IL‐1β increased SGLT1 expression and decreased survival rates of cells. Both mizagliflozin and phlorizin increased the survival rates of IL‐1β‐treated PC12HS cells. These results suggest that mizagliflozin and phlorizin can improve vascular cognitive impairment through the inhibition of neural SGLT1 and phlorizin also does so through the improvement of CBF in a mouse model of small vessel disease.

## Graphical abstract

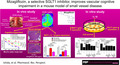

**Abbreviations**
SGLT1: sodium/glucose cotransporter 1
ACAS: asymmetric common carotid artery surgery
CBF: cerebral blood flow
VCI: vascular cognitive impairment
CCA: common carotid artery
GLUT1: glucose transporter 1
GLUT3: glucose transporter 3
MCP‐1: monocyte chemotactic protein 1
IL‐1β: interleukin‐1β
TNF‐α: tumor necrosis factor α

## 1 INTRODUCTION

One of the major public health concerns is aging‐related cognitive impairment associated with alteration in cerebral blood vessels (i.e., vascular cognitive impairment, VCI) in recent years.^1^ Several types of vascular dementia, such as post‐stroke dementia, subcortical ischemic vascular dementia, multi‐infarcted cortical dementia, and mixed dementia, have been reported.^2^, ^3^ At present, VCI is mainly prevented by treating vascular diseases and other risk factors for VCI. In fact, the management of cardiovascular risk factors, such as hypertension, diabetes, dyslipidemia, obesity, and smoking, greatly reduces the development of VCI in later life. The effective control of cardiovascular risk factors prevents VCI‐related disease and may be more effective than current pharmacological treatment for VCI.^4^, ^5^ However, currently available therapeutic options are limited for directly improving vascular dementia and cognitive impairment through protection against neural injury.

Several recent studies showed that mid‐cerebral artery occlusion induced cerebral neuronal damage through sodium‐glucose cotransporter 1 (SGLT1) activation in mice.^6^, ^7^ Recently, a new mouse model of small vessel disease with cognitive impairment, generated by surgical implantation of an ameroid constrictor in the right common carotid artery (CCA) and placement of a microcoil in the left CCA, was developed.^8^ Our previous study demonstrated that SGLT1 participated in the development of VCI and neural injury in the mouse model described above.^9^ These results suggest that blockade of SGLT1 can improve vascular dementia and cognitive impairment through protection against neural injury. However, whether SGLT1 inhibitors are effective for inhibiting neural injury, VCI, and dementia during small vessel disease is still unknown. Mizagliflozin is a new selective SGLT1 blocker and is known to exhibit potential in the amelioration of chronic constipation.^10^ Moreover, mizagliflozin shows favorable efficacy and safety for patients with functional constipation and whose risk of hypoglycemia is low.^11^ As several glucose transporters (GLUTs) such as GLUT 1‐6 exist and function in the brain, the possibility of GLUTs different from SGLT1 participating in the development of VCI and ischemic neuronal injury cannot be denied. Phlorizin is a non‐selective SGLT1 blocker that improves mid‐cerebral artery occlusion‐induced cerebral neuronal damage in mice.^6^, ^7^ Here, we examined the effects of mizagliflozin on ischemia‐induced VCI and neural injury in a mouse model of small vessel disease and compared them with those of phlorizin.

## 2 MATERIALS AND METHODS

### 2.1 Experimental animals and asymmetric common carotid artery stenosis surgery (ACAS)

**FIGURE 1:**
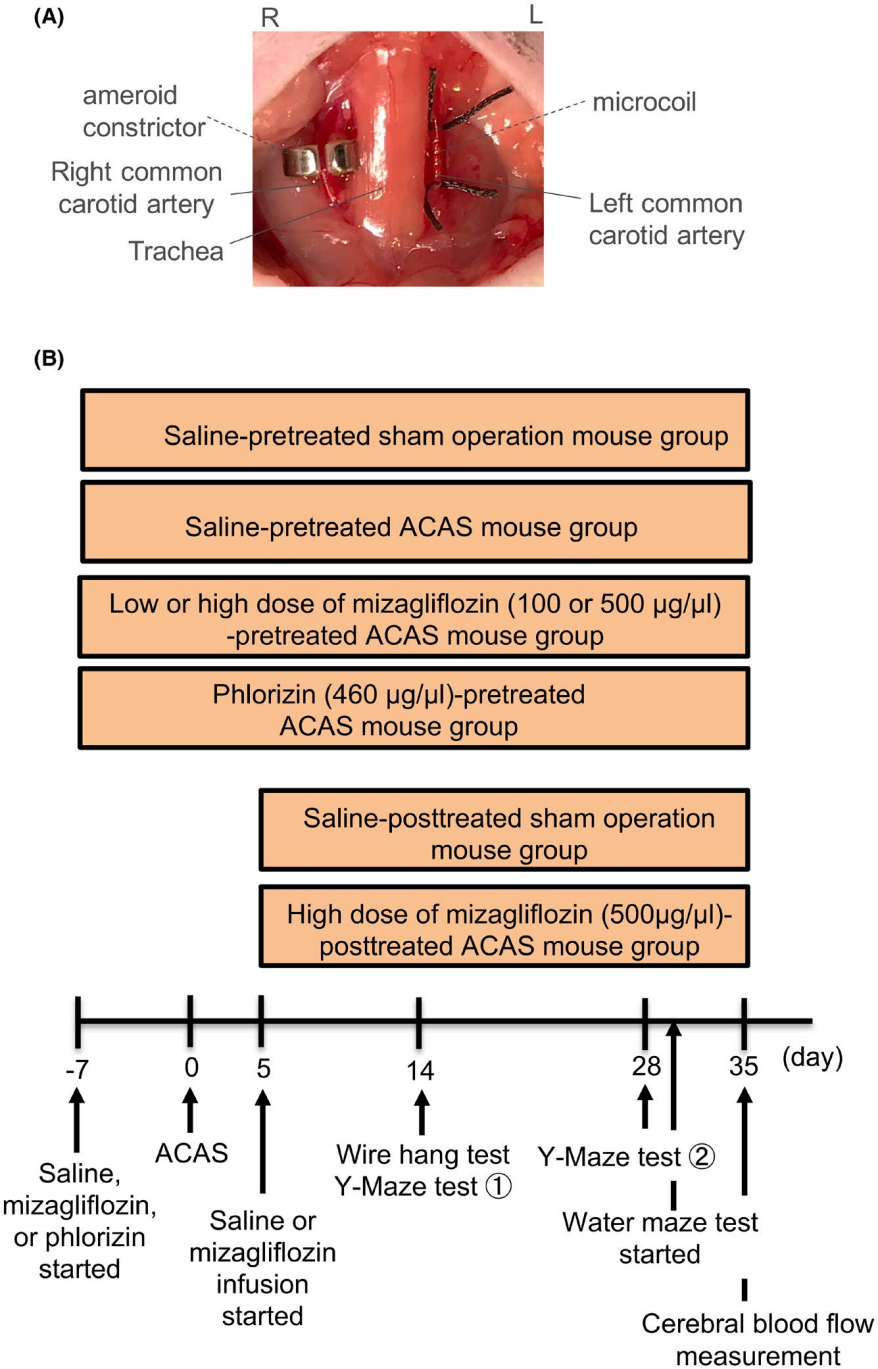
Panel A, Asymmetric common carotid artery surgery (ACAS) was performed in four of the five groups. An image showing an ameroid constrictor (AC) around the right common carotid artery (CCA) and placement of a microcoil around the left CCA. Panel B, The schema of experimental protocols for each of the seven groups (saline‐pretreated sham operation mice, saline‐pretreated ACAS mice, low or high dose of mizagliflozin‐pretreated ACAS mice, phlorizin‐pretreated ACAS mice, saline‐posttreated sham operation mice, and high dose of mizagliflozin‐posttreated ACAS mice). Saline, mizagliflozin (100 or 500 μg/μl), or phlorizin (460 μg/μl) was administered for 42 or 30 days using an osmotic mini pump (Alzet 2006 [0.15 µl/h, 200 µl]) implanted subcutaneously in all mice one week before or five days after sham operation and ACAS, respectively

C57BL/6J male mice (10 weeks old, 25–30‐g body weight) were purchased from Japan SLC. To produce VCI, stenosis of both common carotid arteries (CCAs) was surgically created using a microcoil and an ameroid constrictor (ACAS), as described elsewhere.^8^, ^9^ Briefly, after mice were anesthetized with isoflurane (2–4%), both CCAs were exposed and freed from their sheaths. A spiral microcoil was placed around the left CCA just below the carotid bifurcation. An ameroid constrictor^12^ was also placed around the right CCA (Figure 1A). The same procedure was performed except for ACAS in sham‐operated mice. Finally, we closed the midline cervical incision using 7‐0 nylon suture. After the operation, mice were maintained in an environment with day/night reversal throughout the experiments. Sodium pentobarbital (30 mg/kg) application into abdominal cavity and/or isoflurane inhalation was performed to anesthetize all animals before the brain tissue was excised. The degree of motion of the sternum and movement of the extremities was monitored to confirm the adequacy of anesthesia. The appropriate depth of anesthesia was checked by a toe pinch reflex test. An animal glucometer (LAB Gluco, Fore Care Tokyo Co., Ltd.) was used to measure the blood glucose (BG) level.

### 2.2 Experimental groups and drug administration

Male mice were divided into seven groups (sham‐operated saline‐pretreated mice, saline‐pretreated ACAS mice, low or high doses of mizagliflozin‐pretreated ACAS mice, phlorizin‐pretreated ACAS mice, sham‐operated saline‐posttreated mice, and high doses of mizagliflozin‐posttreated ACAS mice) (Figure 1B). Saline, mizagliflozin (100 or 500 μg/μl, IC_50_ = 166 nM Rat SGLT1), or phlorizin (460 μg/μl) was administered for 42 or 30 days using an osmotic mini pump (Alzet 2006 (0.15 µl/h, 200 µl)) implanted subcutaneously in all mice one week before or five days after sham operation and ACAS, respectively (Figure 1B). We measured the mean plasma mizagliflozin concentration (100 μg/μl; 460 ± 159 nM, 500 μg/μl; 1700 ± 300 nM), suggesting that the dosage of mizagliflozin used in this study was sufficient to inhibit SGLT1.

### 2.3 Wire hang test

The wire hang test was performed at two weeks after ACAS or sham surgery, as described previously.^9^ Briefly, to avoid vibration, the wire was tightly fixed to the top of the box because this might interfere with the performance of the mouse. We placed both upper limbs of a mouse on the metallic wire, and latency to fall was recorded. We repeated the procedure five times with an interval of 5 min between attempts and the mean latency to fall was calculated from the average time of five trials.

### 2.4 Y‐maze test

The Y‐maze test was performed to assess spatial working memory and spontaneous activity, as described elsewhere.^9^, ^13^ Briefly, the Y‐maze test was performed at 2 and 4 weeks after ACAS or sham surgery (Figure 1B). After introduction to the end of the start arm, mice were allowed to freely explore the three arms during an 8‐min session. The numbers of arm entries and trials were recorded using a video camera (Logicool HD Webcam C615, Logicool). A mouse was considered to have entered an arm when all four limbs were within the arm. Spatial working memory was elucidated by the percentage of alternation behavior. Actual alternation behavior was defined as entry into all three arms on consecutive occasions. The maximum alternation behavior was calculated as the total number of arm entries minus two, and the percentage of alternation was calculated as the number of actual alternations divided by that of maximum alternations multiplied by 100. Spontaneous activity was elucidated by the total number of arms entered during a session.

### 2.5 Morris water maze test

The Morris water maze test was performed at 29–34 days after ACAS or sham surgery, as previously described.^9^, ^14^ Briefly, to learn the platform position, a visible platform task was performed using a transparent circular platform (i.e., escape platform) with a diameter of 8 cm three times per day from the first to sixth day. After the visible platform task, we immersed the circular platform 1 cm below the water surface in the center of a quadrant of the pool to perform a hidden platform task. The platform was kept in the same position throughout the experiment. The hidden platform task was performed five times with a 5‐min interval between attempts each day. We released mice into the water at the starting position in three other quadrants. We monitored each mouse tested and recorded the time required until it arrived at the platform. When a mouse arrived at the platform in 90 s, we allowed it to stay there for 15 s. When a mouse did not arrive at the platform in 90 s, we guided it to the platform and stayed it there for 15 s. In each mouse, we calculated latency to reach the platform (escape latency) from the average time of 5 times of the hidden platform task performed each day.

### 2.6 Cerebral blood flow (CBF) measurement

**FIGURE 2:**
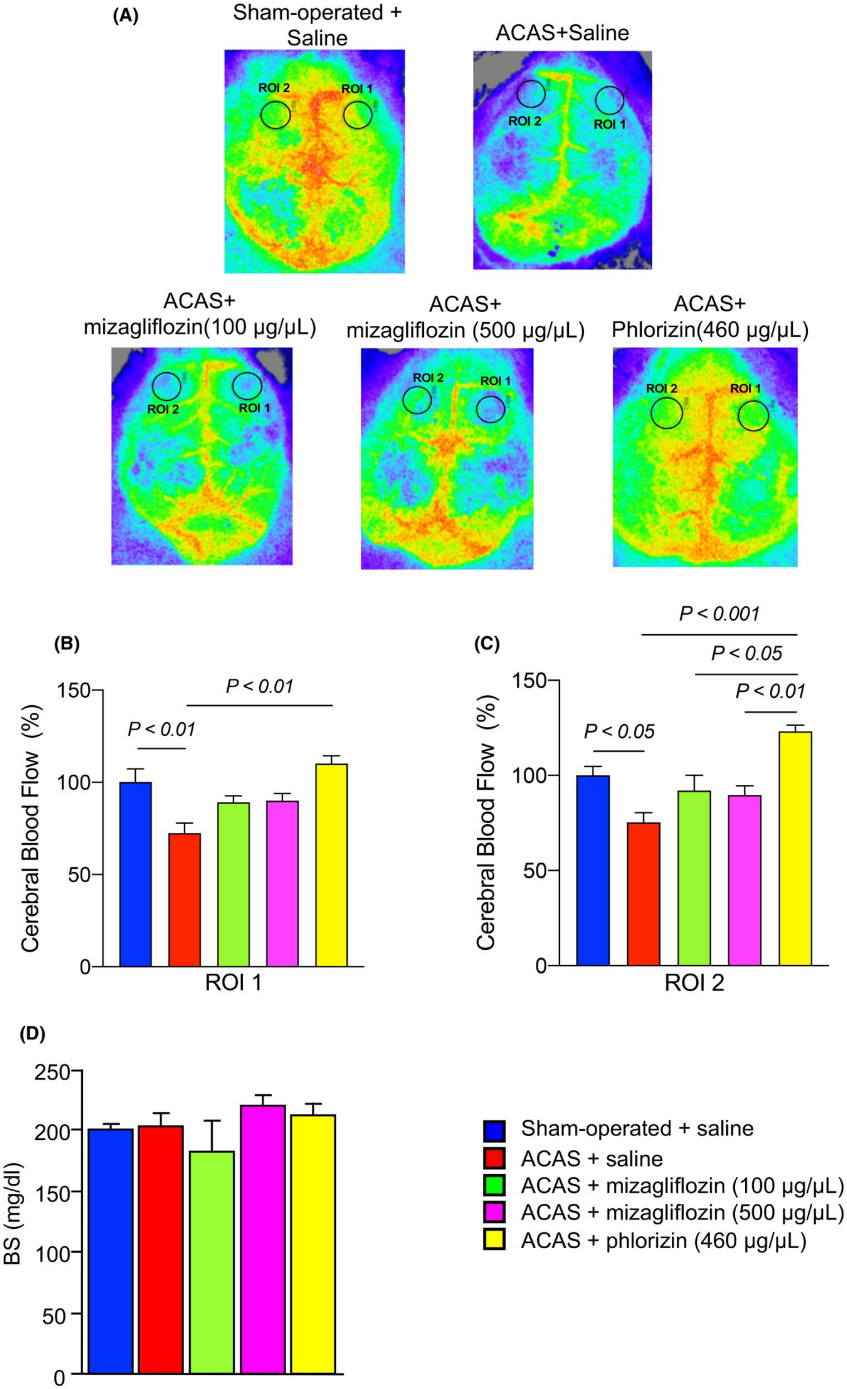
Cerebral blood flow (CBF) and blood sugar (BS) levels in the five different groups of mice at 35 days after ACAS or the sham operation. Saline, mizagliflozin or phlorizin was stared to administer one week before ACAS using an osmotic mini pump. Panel A, Representative CBF images of laser speckle flowmetry. Panel B‐C, Mean local CBFs in circular regions of ROI1 and ROI2. Panel D, Blood sugar (BS) in the five different groups of mice. Data are the mean ± SE obtained from 8 mice for each group. Sham‐operated + saline, saline‐pretreated sham operation mice; ACAS + saline, saline‐pretreated ACAS mice; ACAS + mizagliflozin (100 μg/μl), low dose (100 μg/μl) of mizagliflozin‐pretreated ACAS mice; ACAS + mizagliflozin (500 μg/μl), high dose (500 μg/μl) of mizagliflozin‐pretreated ACAS mice; ACAS + phlorizin (460 μg/μl), phlorizin (460 μg/μl)‐pretreated ACAS mice

We measured CBF at 35 days after ACAS or sham surgery using a 2D laser blood flow imager (OMEGAZONE, Omegawave Inc.), as previously described.^8^, ^9^ Briefly, laser speckle flowmetry with the average of highspeed 2D imaging was used to measure relative CBF. After anesthesia (1–2% isoflurane), we surgically removed the mouse scalp to expose the skull. The periosteum, which adheres to the skull, was widely removed with fine‐tip forceps. The skull surface was wiped with saline‐soaked gauze to prevent drying of the surface. Cerebral blood flow recordings were performed through the skull. Circular regions in identically sized ROIs (900 pixels), located 1 mm posterior and 2 mm lateral to the bregma were defined on the image for quantitative measurement (Figure 2A).

### 2.7 Quantification of mRNA by real‐time PCR

The amount of mRNA by real‐time reverse transcription‐polymerase chain reaction (RT‐PCR) was performed as previously described.^15^ Briefly, after the brain tissue was resected, total RNA was prepared from the brain tissue of the four groups of mice (Figure 1B, *n* = 6 for each) using ReliaPrep^TM^ RNA Tissue Miniprep System (Promega), according to the manufacturer's instructions. We used 500 ng of total RNA as a template for reverse transcription with the SuperScript^®^ III First‐Strand synthesis system (Invitrogen). We performed real‐time RT‐PCR analysis with an ABI Step One Real‐Time PCR System using Fast SYBR Green Master Mix (Applied Biosystems) to detect SGLT1, glucose transporter 1 and 3 (GLUT1 and 3), monocyte chemotactic protein 1 (MCP‐1), interleukin‐1β (IL‐1β), tumor necrosis factor α (TNF‐α), and glyceraldehyde‐3‐phosphate dehydrogenase (GAPDH). Table 1 showed the sequences of oligonucleotide primers. As the expression of GAPDH mRNA was constant between groups the expression of each gene was normalized to that of GAPDH mRNA.

**TABLE 1:** Sequences of oligonucleotide primers

Primer		Araay
GAPDH	S	TGTGTCCGTCGTGGATCTGA
	A	TTGCTGTTGAAGTCGCAGGAG
GLUT1	S	TCAACACGGCCTTCACTG
	A	CACGATGCTCAGATAGGACATC
GLUT3	S	TTCTGGTCGGAATGCTCTTC
	A	AATGTCCTCGAAAGTCCTGC
IL‐1β	S	TCCAGGATGAGGACATGAGCAC
	A	GAACGTCACACACCAGCAGGTTA
TNF‐α	S	AAGCCTGTAGCCCACGTCGTA
	A	GGCACCACTAGTTGGTTGTCTT
SGLT‐1	S	TTGGAGTCCTCTGGGATGTC
	A	GCCATCATCCTCTTCGTCAT
MCP‐1	S	GGCTCAGCCAGATGCAGTTAAC
	A	GCCTACTCATTGGGATCATCTTG

### 2.8 Histology

Hematoxylin/eosin staining for histopathological analysis was performed as previously described.^9^ Briefly, after anesthesia with isoflurane (2%), and sodium pentobarbital (15 mg/kg i.p.) we transcardially perfused and fixed mouse brain with a 4% solution of paraformaldehyde in 0.01 mol/L PBS for more than 5–8 min in the four different groups of mice (shown in Figure 1B, *n* = 6 for each). After brain was fixated, we quickly removed each brain and divided it coronally at the bregma, the bregma 1 mm, and bregma 2 mm through the hippocampus. were We embedded the brain samples in paraffin and then cut them into 3‐μm‐thick coronal sections. For histopathological analysis, six sections stained with hematoxylin/eosin were performed were prepared. After HE staining, the shrunken neurons with pyknotic nuclei were counted to assess neuropathy.

### 2.9 Cell Culture, Hypoxia, and IL‐1β Stimulation

Rat PC12HS cells (JCRB0266, Japanese Cancer Research Bank, National Institutes of Biomedical Innovation) were seeded on collagen type I‐coated 6‐well plates (1 × 10^6^ cells/well) and maintained in “complete medium” that consisted of RPMI1640 medium supplemented with 5% fetal bovine serum (FBS) and 10% horse serum) in a humidified incubator at 37℃, 5% CO_2_, for 24 h. Then, the cells were incubated in 50 ng/ml of nerve growth factor (mouse NGF 2.5S, N‐240, Alomone Labs, Jerusalem, Israel)‐added completed medium at 37℃, 5% CO_2_, for 72 h to allow neural differentiation. To examine effects of hypoxia on PC12HS cells, the cells were cultured under hypoxic conditions (1% O_2_/ 5% CO_2_, 94% N_2_) at 37℃ by use of a multi‐gas incubator (MCO‐170MUV‐PJ, Panasonic Healthcare Corp) for 1 h. Thereafter, the media were replaced with complete media containing 1 μM or 10 μM of mizagliflozin or 10 μM phlorizin was added to the culture, and the cells were incubated in an incubator at 37℃, 5% CO_2_ under the normoxic conditions for another 22 h. To examine effects of IL‐1β stimulation on PC12HS cells, the cells were exposed to 100 ng/ml rat IL‐1β (CYT‐395, ProSpec) for 48 h. Mizagliflozin or phlorizin at a concentration of 1 or 10 μM was added to the culture 24 h before or 6 h after IL‐1β stimulation started. After hypoxia or the IL‐1β stimulation, the cells were harvested and homogenized in Sepasol‐RNA I Super G (Nacalai Tesque), and total RNAs were extracted according to the manufacture's instructions. After treatment with DNase (Nippon Gene), cDNAs were synthesized with Moloney Murine Leukemia Virus reverse transcriptase (Nippon Gene) and used for real‐time PCR with GeneAce SYBR qPCR Mix α Low ROX (Nippon Gene). Real‐time RT‐PCR was performed with ABI Step One Real‐Time PCR System using Fast SYBR Green Master Mix (Applied Biosystems) to detect SGLT1 and MCP‐1. The sequences of oligonucleotide primers used in this experiment are shown in Table 1. The Ct values of the target genes were normalized with the corresponding Ct value of eukaryote translation elongation factor 1 α (EF1α), and the relative expression level was estimated by 2^−ΔΔCt^ methods.^16^ PC12HS cell viability was measured using Cell Counting Kit‐8 (Dojindo, Kumamoto, Japan) according to the manufacturer's instructions.

### 2.10 Drugs

Mizagliflozin (3‐(3‐{4‐[3‐(β‐D‐glucopyranosyloxy)‐5‐isopropyl‐1*H*‐pyra zol‐4‐ylmethyl]‐3‐methylphenoxy}propylamino)‐2,2‐dimethylpropionamide) is a selective SGLT1 blocker, which was kindly provided by Kissei Pharmaceutical Co., Ltd. Phlorizin dihydrate was purchased from Sigma‐Aldrich Co.

### 2.11 Statistical analysis

All data are shown as the mean ± SE. We used an analysis of variance (ANOVA) with Dunnett's test for the statistical analysis of multiple comparisons of data. Two‐way and one‐way ANOVA with Dunnett's test were used for statistical analysis of the Morris water maze test and other experiments, respectively. *p* < .05 was considered significant.

### 2.12 Nomenclature of targets and ligands

Key protein targets and ligands in this article are hyperlinked to corresponding entries in http://www.guidetopharmacology.org, the common portal for data from the IUPHAR/BPS Guide to PHARMACOLOGY,^17^ and are permanently archived in the Concise Guide to PHARMACOLOGY 2019/20.^18^

## 3 RESULTS

### 3.1 Mizagliflozin did not reverse ACAS‐induced decreases in CBF

To examine the effects of mizagliflozin on ACAS‐induced decreases in CBF, CBF was measured and local CBFs were calculated in circular regions of ROI1 and ROI2 (Figure 2A). Representative images of laser speckle flowmetry revealed that CBF was decreased in saline‐treated ACAS compared with sham operation mice (Figure 2A). CBF was similar between saline‐treated and mizagliflozin‐treated ACAS mice. Interestingly, CBF was similar between sham‐operated and phlorizin‐treated ACAS mice. Quantitative analysis demonstrated that ACAS decreased local CBF in both circular regions of saline‐treated ACAS compared with sham operation mice (Figure 2B and C). Mizagliflozin did not reverse the decreased local CBF in ACAS mice regardless of the infusion concentration. In contrast, phlorizin significantly reversed the decreased local CBF in both circular regions of ACAS mice (Figure 2B and C).

### 3.2 Mizagliflozin and phlorizin did not affect blood glucose levels

Blood glucose levels were similar among the five different groups of mice (Figure 2D).

### 3.3 Mizagliflozin reversed ACAS‐induced shortening of latency to fall measured by wire hang test

**FIGURE 3:**
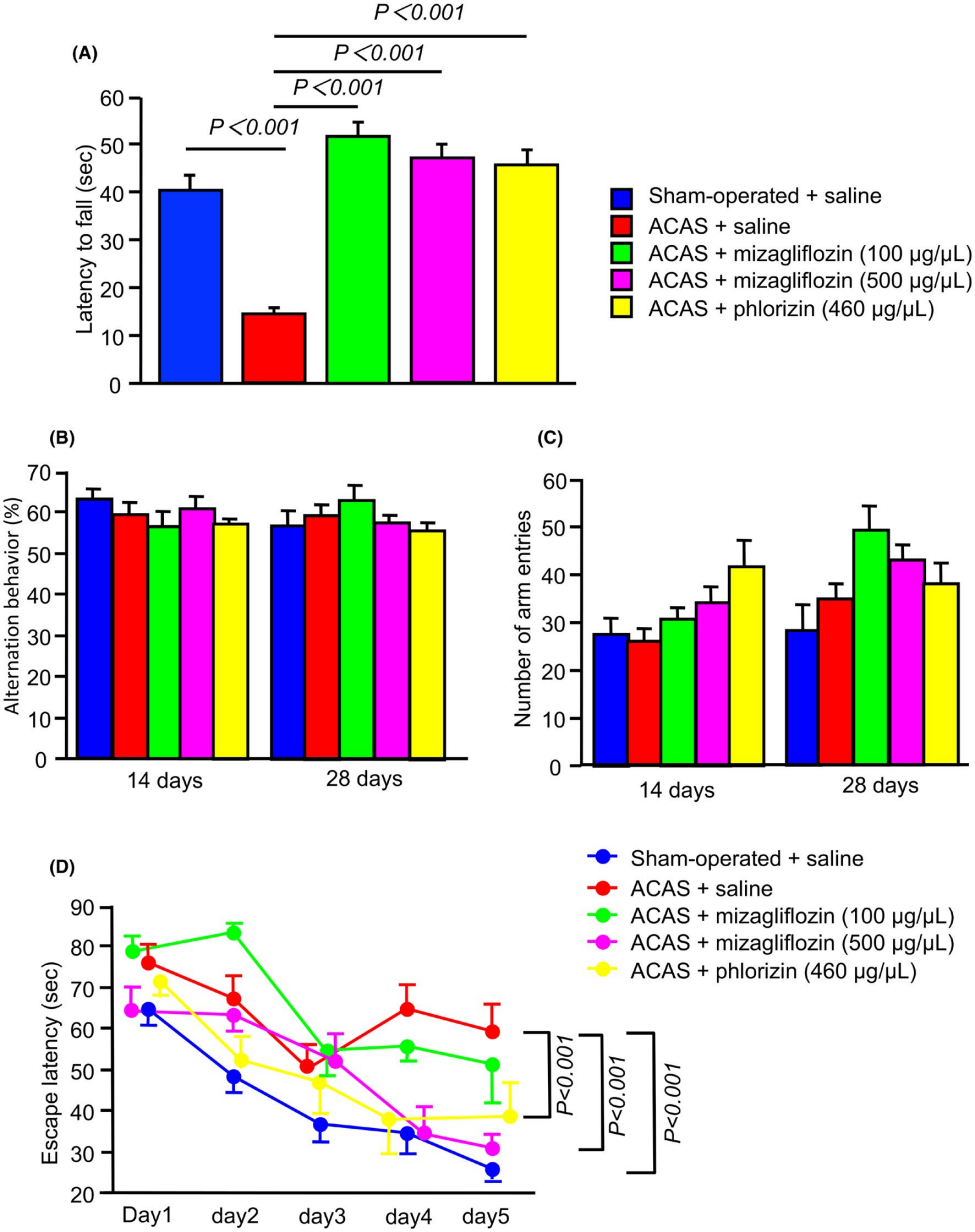
Behavioral performance of ACAS mice in wire hang, Y‐maze, and Morris water maze tests. Saline, mizagliflozin or phlorizin was stared to administer one week before ACAS using an osmotic mini pump. Panel A, Neuromuscular strength elucidated by latency to fall assessed with the wire hang test 14 days after ACAS or the sham operation in the five different groups of mice. Panel B‐C, Spatial working memory elucidated by alternation behavior and spontaneous activity elucidated by the number of arm entries on the Y‐maze test at 14 and 28 days after ACAS or the sham operation in the five different groups of mice. Panel D, Time‐course of escape latency assessed with the Morris water maze test from days 1 to 6 (from 29 to 34 days after ACAS or the sham operation) in the five different groups of mice. Data are the mean ± SE obtained from 8 mice for each group

The wire hang test showed that the latency to fall was shorter in saline‐treated ACAS compared with sham‐operated mice (Figure 3A). Moreover, ACAS significantly shortened the latency to fall in saline‐treated ACAS mice compared with mizagliflozin‐treated or phlorizin‐treated ACAS mice. (Figure 3A). Interestingly, the latency to fall was similar among sham‐operated mice, low or high concentrations of mizagliflozin‐treated ACAS mice, and phlorizin‐treated ACAS mice, indicating that mizagliflozin and phlorizin can improve the shortened latency to fall to the normal level in ACAS mice (Figure 3A).

### 3.4 Mizagliflozin did not affect spatial working memory or spontaneous activity measured by Y‐maze test

The Y‐maze test showed that the percentage of alternation behaviors was similar among the five different groups of mice at two and four weeks after ACAS (Figure 3B). Moreover, the rates of arm entries were also similar among the five different groups of mice at two and four weeks after ACAS (Figure 3C).

### 3.5 Mizagliflozin reversed ACAS‐induced prolongation of escape latency measured by Morris water maze test

The Morris water maze test showed that the escape latency was significantly longer in saline‐treated ACAS mice than in sham‐operated mice during the acquisition phase of 5 days. High concentrations of mizagliflozin and phlorizin significantly shortened the prolonged escape latency in ACAS mice during the acquisition phase of 5 days (Figure 3D). In contrast, a low concentration of mizagliflozin failed to shorten the prolonged escape latency in ACAS mice, suggesting that mizagliflozin at a high concentration and phlorizin reverse the development of spatial learning and reference memory impairment induced by ACAS. Mean swimming speeds in the acquisition phase did not differ among the five different groups of mice (data not shown).

### 3.6 Mizagliflozin ameliorated increase in number of shrunken neurons in ACAS mice

**FIGURE 4:**
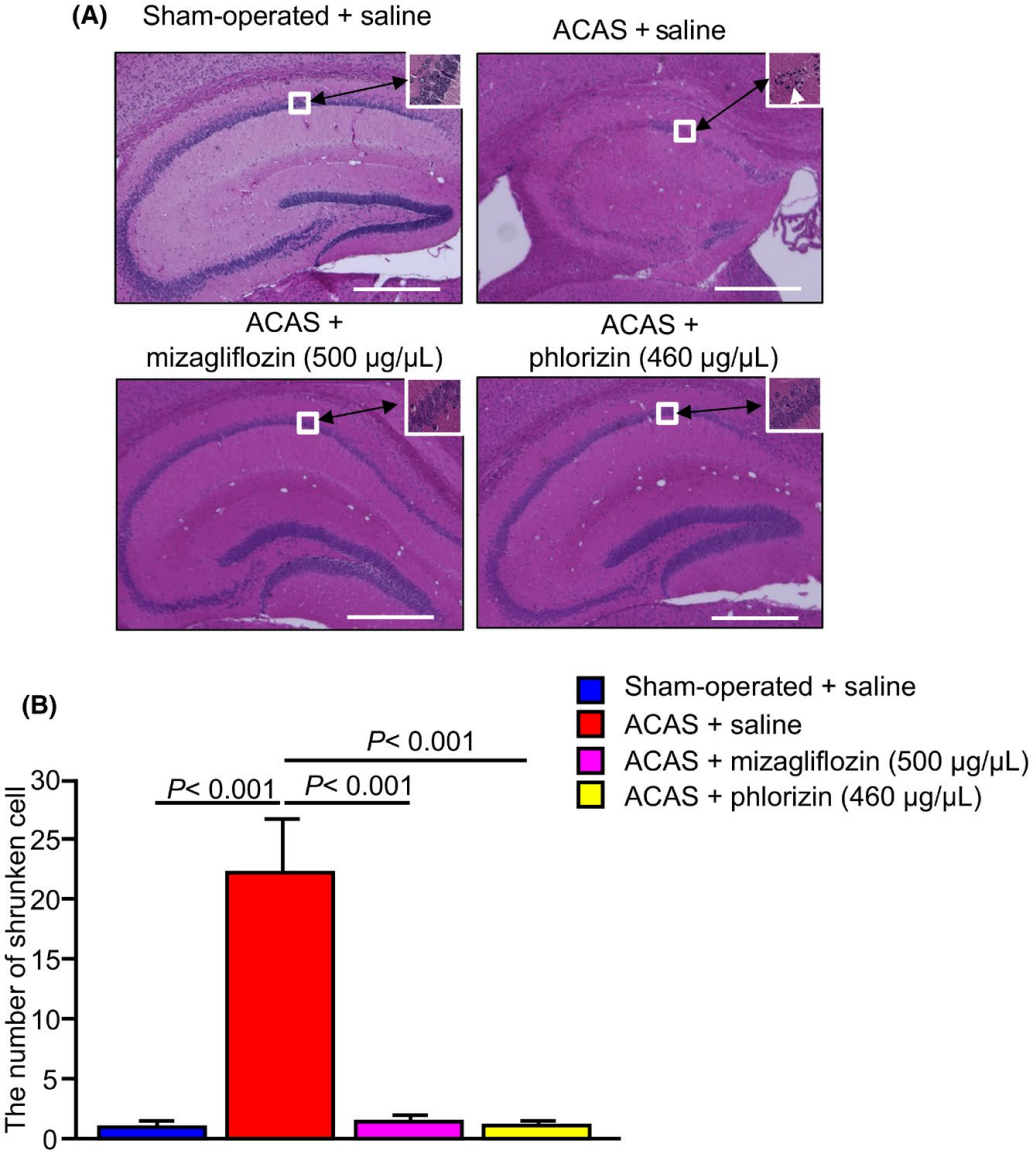
Historical analysis of ischemia‐induced neuronal injury in the mouse hippocampus in the four different groups of mice at 35 days after ACAS or the sham operation. Saline, mizagliflozin or phlorizin was stared to administer one week before ACAS using an osmotic mini pump. Panel A, Typical image of HE staining (Original magnification: 2 × Scale bar = 500 μm). White arrow shows shrunken neurons with pyknotic nuclei. Panel B, Quantitative analysis of shrunken neurons with pyknotic nuclei calculated from neurons located within the white square area shown in panel A

To examine the effects of mizagliflozin on ischemia‐induced neuronal damage, shrunken neurons with pyknotic nuclei in the hippocampal area were observed. Representative images of the hippocampal area revealed pyknotic cell death (white arrows) in saline‐treated ACAS mice but not among the three other groups of mice (Figure 4A). Quantitative analysis of shrunken neurons with pyknotic nuclei in the C1 area of the hippocampus showed an increased number of shrunken neurons in saline‐treated ACAS mice compared with the three other groups of mice (Figure 4B).

### 3.7 Mizagliflozin did not reduce IL‐1β and TNF‐α mRNA expressions in ACAS mice

**FIGURE 5:**
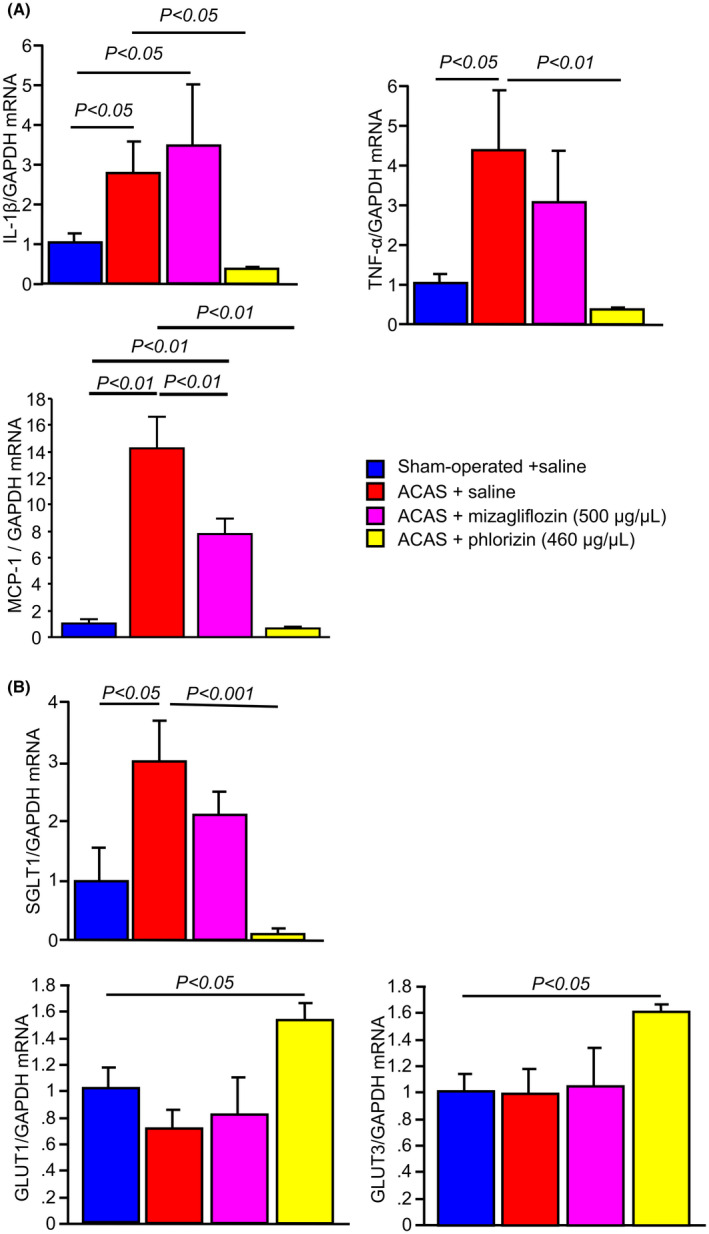
Quantitative analyses of pro‐inflammatory cytokines, a chemokine, and glucose transporters gene expressions by real‐time RT‐PCR in the four different groups of mouse brain tissues at 35 days after ACAS or the sham operation. Saline, mizagliflozin or phlorizin was stared to administer one week before ACAS using an osmotic mini pump. Panel A, mRNA levels of monocyte chemotactic protein 1 (MCP‐1), interleukin 1β (IL‐1β), and tumor necrosis factor α (TNF‐α) are shown. Panel B, mRNA levels of SGLT1, GLUT1, and GLUT3 are shown. Data for SGLT1, GLUT1, GLUT3, MCP‐1, IL‐1β, and TNF‐α were normalized to those for GAPDH. Data are the mean ± SE obtained from 6 mice for each group. mRNA

mRNA expressions of IL‐1β and TNF‐α genes were significantly increased in saline‐treated ACAS compared with sham‐operated mouse brains. Saline‐treated ACAS mice had an increase in IL‐1β and TNF‐α mRNA expression which was prevented by phlorizin treatment. Mizagliflozin failed to block this increase (Figure 5A upper). Moreover, expression of MCP‐1 mRNA was significantly increased in saline‐treated ACAS compared with sham‐operated mouse brains (Figure 5A lower). Mizagliflozin reduced the increased mRNA expression of the MCP‐1 gene in ACAS mouse brains. However, it was still significantly increased in mizagliflozin‐treated ACAS compared with sham‐operated mice (Figure 5A lower). In contrast to mizagliflozin, phlorizin reduced the increased mRNA expression of the MCP‐1 gene and normalized it in ACAS mouse brains (Figure 5A lower).

### 3.8 Mizagliflozin did not reduce increased SGLT1 mRNA expression in ACAS mice

mRNA expression of SGLT1 was significantly increased in saline‐treated ACAS compared with sham‐operated mouse brains (Figure 5B upper). Mizagliflozin did not reduce the increased SGLT1 gene expression in ACAS mouse brain. Interestingly, phlorizin significantly ameliorated the increased SGLT1 gene expression in ACAS mouse brain. Moreover, phlorizin but not mizagliflozin increased GLUT1 and GLUT3 gene expressions in ACAS compared with sham‐operated mouse brains (Figure 5B lower).

### 3.9 Post treatment with mizagliflozin induced prolongation of escape latency measured by Morris water maze test in ACAS mice

**FIGURE 6:**
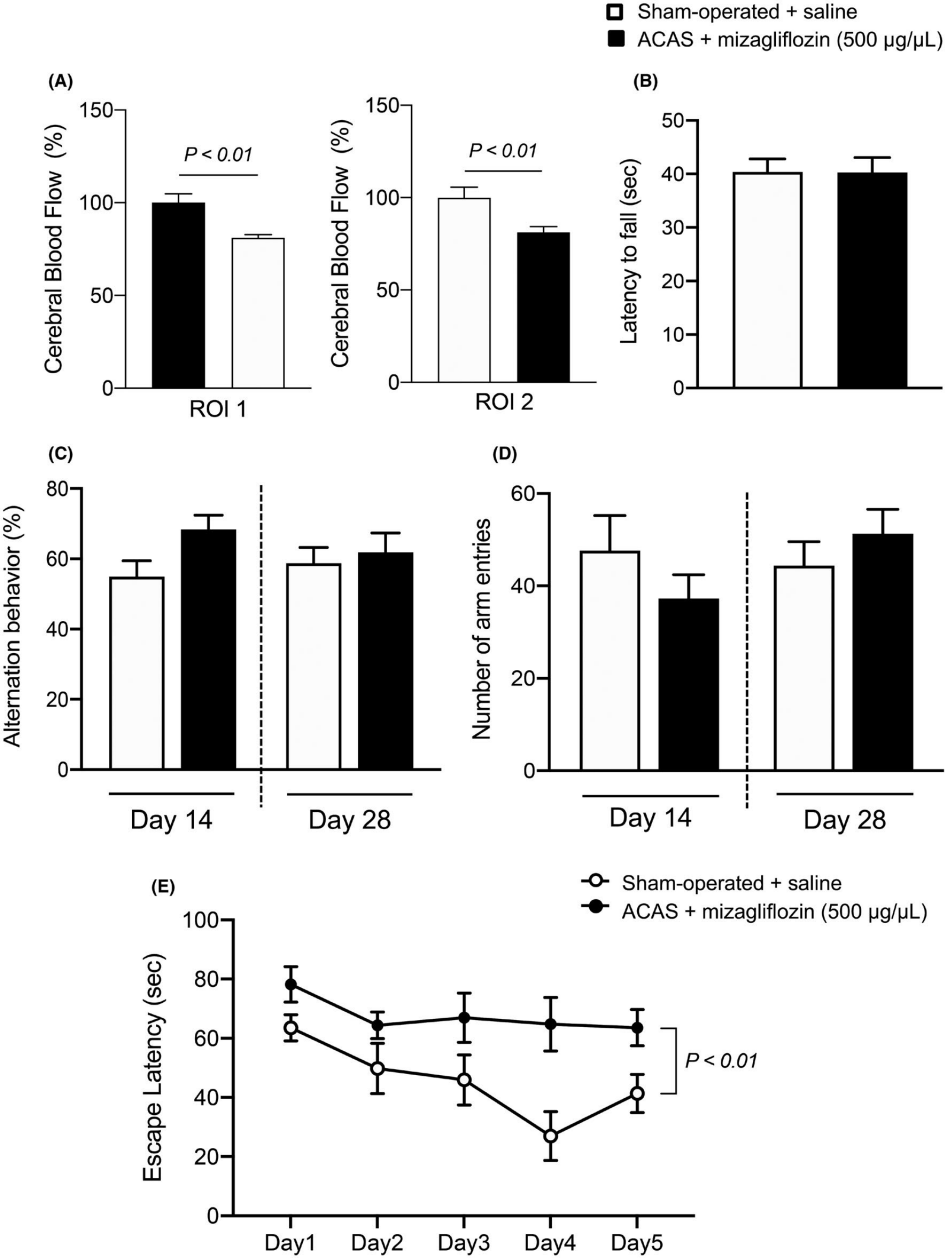
CBF and behavioral performance in mizagliflozin‐posttreated ACAS and saline‐posttreated sham operated mice. Saline or mizagliflozin began to be subcutaneously infused 5 days after ACAS using an osmotic mini pump. Panel A, Mean local CBFs in circular regions of ROI1 and ROI2 at 35 days after ACAS or the sham operation in the two different groups of mice. Panel B, Neuromuscular strength elucidated by latency to fall assessed with the wire hang test 14 days after ACAS or the sham operation in the two different groups of mice. Panels C and D, Spatial working memory elucidated by alternation behavior, and spontaneous activity elucidated by the number of arm entries on the Y‐maze test at 14 and 28 days after ACAS or the sham operation in the two different groups of mice. Panel E, Time‐course of escape latency assessed with the Morris water maze test from days 1 to 6 (from 29 to 34 days after ACAS or the sham operation) in the two different groups of mice. Data are the mean ± SE obtained from 8 mice for each group. Sham‐operated + saline, saline‐posttreated sham operation mice; ACAS + mizagliflozin (500 μg/μl), high dose (500 μg/μL) of mizagliflozin‐posttreated ACAS mice

We examined the effects of post‐treatment with mizagliflozin (5 days after ACAS) on behavioral performance in ACAS mice. CBF was decreased in ACAS mice receiving post‐treatment with mizagliflozin compared with sham‐operated mice (Figure 6A). The latency to fall measured by the wire hang test was similar between mizagliflozin‐treated ACAS and sham‐operated mice (Figure 6B). The Y‐maze test showed that the percentage of alternation behaviors and rates of arm entries were similar between mizagliflozin‐treated ACAS and sham‐operated mice (Figure 6C). In contrast, the Morris water maze test showed that the escape latency was significantly longer in mizagliflozin‐treated ACAS mice than in sham‐operated mice during the 5‐days acquisition phase (Figure 6D).

### 3.10 Both mizagliflozin and phlorizin prevented and improved IL‐1β‐induced cell injury in PC12HS cells

**FIGURE 7:**
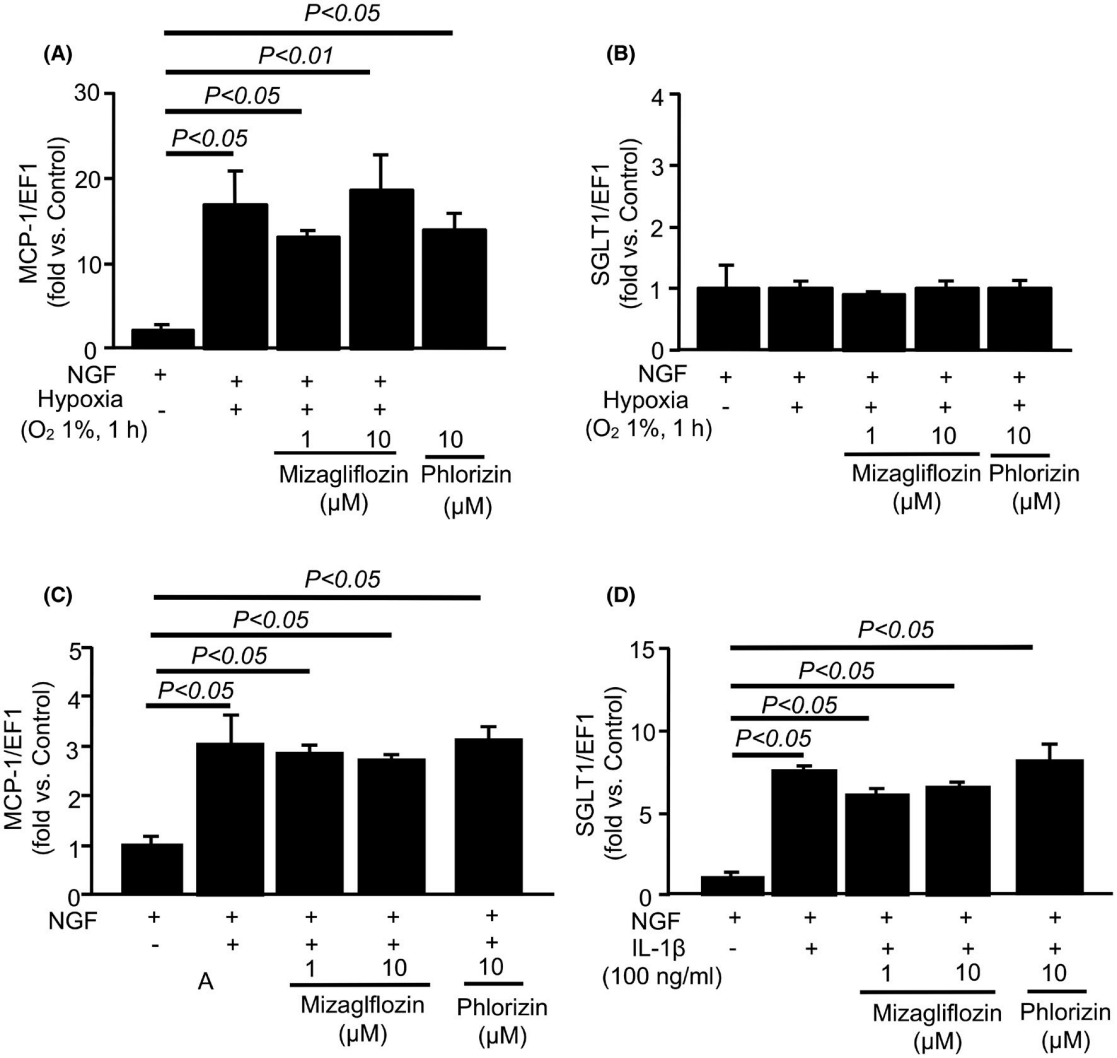
Quantitative analyses of SGLT1 and MCP‐1 gene expressions by real‐time RT‐PCR. Panels A and B, mRNA levels of MCP‐1 (A) and SGLT1 (B) in hypoxia‐stimulated PC12HS cells are shown. Mizagliflozin or phlorizin at a concentration of 1 or 10 μM was added to the culture 1 h after hypoxia started. Panels C and D, mRNA levels of MCP‐1 (C) and SGLT1 (D) are shown in IL‐1β‐stimulated PC12HS cells. Mizagliflozin or phlorizin at a concentration of 1 or 10 μM was added to the culture 24 h before IL‐1β stimulation started. MCP‐1 and SGLT1 were normalized with the corresponding Ct value of eukaryote translation elongation factor 1 α (EF1α)

**FIGURE 8:**
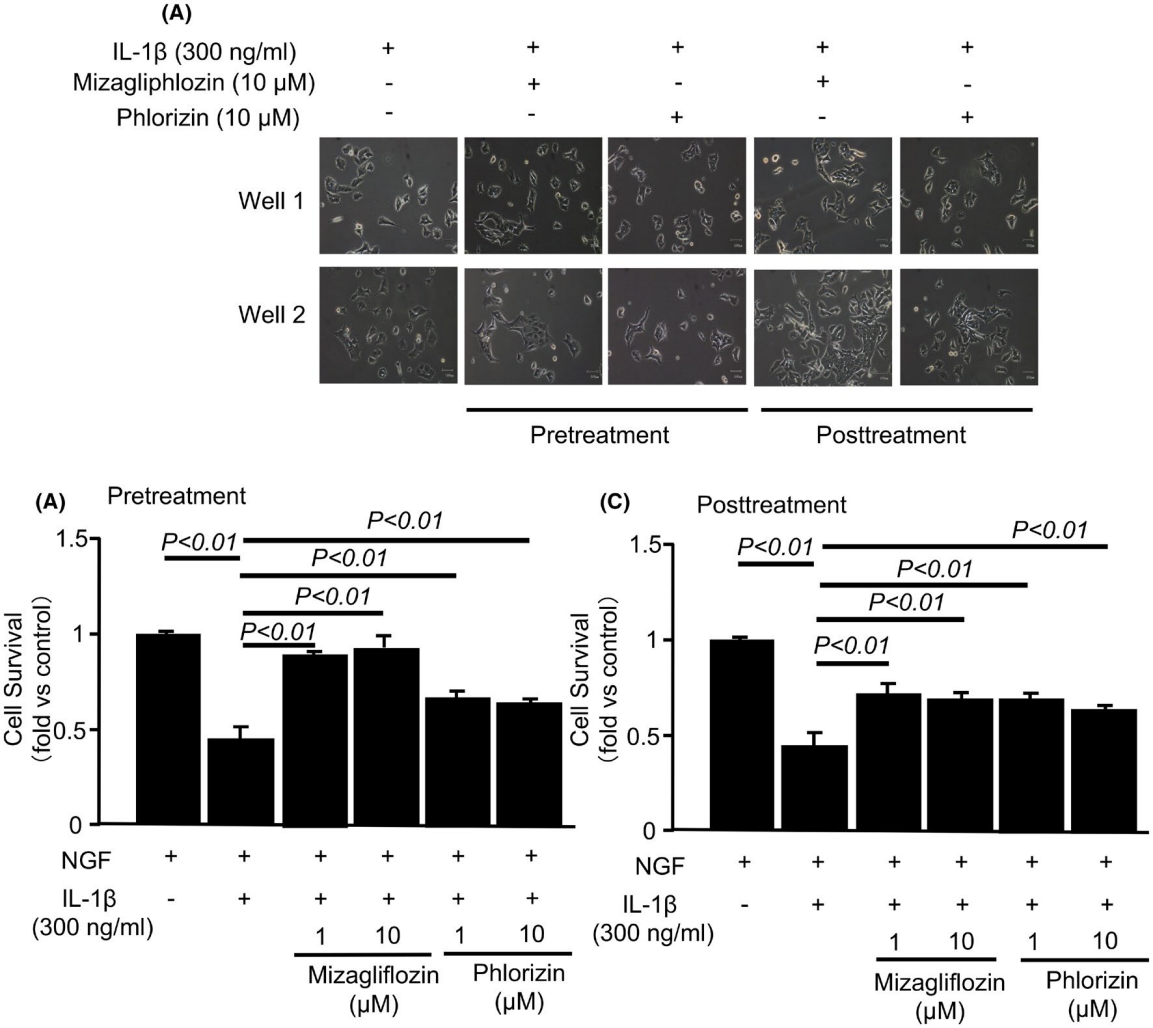
Survival rates of IL‐1β‐stimulated PC12HS cells. Panels A, Representative example of IL‐1β‐stimulated PC12HS cells, when mizagliflozin or phlorizin at a concentration of 1 or 10 μM was added to the culture 24 h before (pretreatment) or 6 h after (posttreatment) IL‐1β stimulation started. Panels B and C, The survival of IL‐1β‐stimulated PC12HS cells when mizagliflozin or phlorizin at a concentration of 1 or 10 μM was added to the culture 24 h before (B, pretreatment) or 6 h after (C, posttreatment) IL‐1β stimulation started. The Cell Counting Kit‐8 assay was used to analyze the survival rate of PC12HS cells. Data are the mean ± SE obtained from 4 wells for each group

To examine the mechanism of mizagliflozin‐ and phlorizin‐induced protection against vascular cognitive impairment and neural damage, we examined the effects of mizagliflozin on hypoxia‐induced SGLT1 and MCP‐1 gene expressions in PC12HS cells. Hypoxia increased mRNA expressions of MCP‐1 but not SGLT1, and mizagliflozin did not reduce hypoxia‐induced increases in MCP‐1 gene expression in PC12HS cells (Figure 7A and B). We also examined the effects of mizagliflozin and phlorizin on SGLT1 and MCP‐1 gene expressions in IL‐1β‐treated PC12HS cells. IL‐1β increased mRNA expressions of SGLT1 and MCP‐1, and neither mizagliflozin nor phlorizin reduced their increased gene expressions in PC12HS cells (Figure 7C and D). Moreover, we examined the effects of mizagliflozin or phlorizin on cell death in IL‐1β‐treated PC12HS cells. Representative images of PC12HS cells show that IL‐1β decreased the number of living cells (Figure 8A). Both Pre‐ and Post‐treatment with mizagliflozin and phlorizin prevented cell death in IL‐1β‐treated PC12HS cells (Figure 8A). The Cell Counting Kit‐8 assay demonstrated that IL‐1β decreased the survival rate of PC12HS cells. Both mizagliflozin and phlorizin ameliorated IL‐1β‐induced decreases in the survival rate of PC12HS cells regardless of the administration start time (Figure 8B and B).

## 4 DISCUSSION

We demonstrated that: 1) mizagliflozin did not ameliorate the decreased cerebral blood flow in ACAS mice, 2) mizagliflozin reversed the shortened latency to fall based on wire hang testing in ACAS mice, 3) pre‐treatment with mizagliflozin reversed the longer escape latencies in ASCS mice based on the Morris water maze test, 4) mizagliflozin improved pyknotic cell death in the hippocampal area of ACAS mice. Even after mizagliflozin treatment, SGLT1, MCP‐1, IL‐1β, and TNF‐α gene expressions significantly increased in ACAS compared with sham‐operated mouse brains. Interestingly, IL‐1β increased SGLT1 and MCP‐1 gene expression in PC12HS cells. Mizagliflozin did not reverse IL‐1β‐induced increases in SGLT1 and MCP‐1 gene expression but improved IL‐1β‐induced cell death in PC12HS cells. These results suggest that mizagliflozin improves VCI and neural injury through an inhibitory action against SGLT1 located on neurons in a mouse model of ACAS‐induced vascular dementia.

Cerebral small vessel disease causes blood–brain barrier (BBB) functional damage, chronic inflammation such as the increased IL‐1β and TNF‐α expression, and leukocyte infiltration, leading to the development of neuron and oligodendrocyte injury.^19^ A study demonstrated that increased IL‐1β and TNF‐α expressions were observed in the hippocampus of a rat cerebral small vessel disease model.^20^ In addition, cerebral hypoperfusion induced a longer escape latency in the Morris water maze test in rats.^21^ Moreover, hypoxia‐inducible factor‐1 (HIF‐1) mediated transcriptional activation of IL‐1β in astrocyte cultures.^22^ Interestingly, SGLT1 existed in neuron and brain small vessel endothelial cells but not in glial cells,^23^, ^24^ and cerebral ischemia increased SGLT1 expression in mouse brains.^6^ Our previous study demonstrated that ACAS‐induced decreases in cerebral blood flow (i.e., cerebral hypoperfusion) caused a longer escape latency based on the Morris water maze test, hippocampal neuronal death, and increased SGLT1, IL‐1β, and TNF‐α gene expressions in WT mice, whereas none of these occurred in SGLT1 knock out mice, suggesting that cerebral hypoperfusion‐induced increases in SGLT1, IL‐1β, and TNF‐α gene expressions play important roles in the development of neural injury and VCI. The present study demonstrated that mizagliflozin, a selective SGLT1 inhibitor, did not reduce the increased gene expressions of SGLT1, IL‐1β, and TNF‐α in ACAS mice. Nevertheless, mizagliflozin reversed the longer escape latency based on the Morris water maze test and hippocampal neuronal death in ACAS mice. Although the differences in the effects of SGLT1 gene deletion with mizagliflozin on ACAS‐induced increases in those gene expressions are still unclear, the increased IL‐1β gene expression might cause neural injury. In fact, Dai et al^25^ demonstrated that BV2 microglia activated by lipopolysaccharide releases IL‐1β, leading to PC12 cell apoptosis. IL‐1β induced MCP‐1 in ARPE‐19 cells through p38MAPK signaling pathways.^26^ Activation of p38MAPK after cerebral ischemia upregulated cerebral SGLT1.^27^ We demonstrated that IL‐1β increased SGLT1 and MCP‐1 gene expressions in PC12HS cells, suggesting that IL‐1β increases SGLT1 gene expression through p38MAPK signaling pathways in PC12HS cells. Mizagliflozin did not reduce IL‐1β‐induced increases in SGLT1 and MCP‐1 gene expressions, whereas it ameliorated IL‐1β‐induced cell death in PC12HS cells. These results suggest that mizagliflozin prevents hippocampal neuronal death, leading to VCI, through an inhibitory action against neuronal SGLT1 in a mouse model of ACAS‐induced vascular dementia, even though mizagliflozin did not improve the increased SGLT1 and IL‐1β gene expressions. However, the mechanism of SGLT1 inhibition in the improvement of neural death and VCI is still uncertain. Previous studies suggested that SGLT1 causes ischemic brain injury, especially simultaneously developed hyperglycemia^6^ via Na^+^ influx through SGLT1.^7^ In fact, SGLT1 allowed sodium ions to flow into the cells, leading to induce cell membrane depolarization and calcium overload.^28^, ^29^ Those changes in neurons were associated with the development of cerebral ischemic neuronal damage.^30^, ^31^ Therefore, cerebral SGLT1 inhibition by mizagliflozin might improve cerebral ischemic neuronal damage through the inhibition of membrane depolarization and calcium overload induced by Na^+^ influx.

In this study, cerebral gene expression of MCP‐1 was increased in ACAS mice. Cerebral ischemia increased MCP‐1 expression in astrocytes, neurons, and endothelial cells.^32^, ^33^ In addition, chronic cerebral hypoperfusion induced MCP‐1‐mediated activation of microglia, white matter lesions, and cognitive deficits in mice.^34^ Moreover, MCP‐1 is likely to increase IL‐1β and TNF‐α expressions in microglia.^35^ Therefore, the cerebral ischemia increases MCP‐1 expression in endothelial cells and/or neurons, which causes the activation of microglia, leading to inflammatory response such as increases in IL‐1β and TNF‐α expression, and VCI in small vessel disease. Also, MCP‐1 can regulate the migration and infiltration of monocytes/macrophages into the brain. and the activated macrophages release several cytokines such as IL‐1β and TNF‐α, leading to the exacerbation of inflammation and neuronal injury. Moreover, MCP‐1 increased brain endothelial permeability through its receptors present on brain endothelial cells.^36^ This study demonstrated that even after mizagliflozin treatment, MCP‐1 gene expressions significantly increased in ACAS compared with sham operation mouse brains. Thus, the mizagliflozin‐induced inhibition of SGLT1 might not inhibit the activation of microglia or infiltration by macrophages, which express and release IL‐1β and TNF‐α. In fact, the present study demonstrated that mizagliflozin did not inhibit the increased gene expressions of IL‐1β and TNF‐α in ACAS mice. In this study, IL‐1β increased mRNA expressions of SGLT1 and MCP‐1, but mizagliflozin did not reduce their increased gene expressions in PC12HS cells (Figure 7A and B). Moreover, hypoxia increased mRNA expressions of MCP‐1 but not SGLT1, but mizagliflozin did not decrease MCP‐1 gene expression in PC12HS cells. These results suggest that neurons recruit macrophages and activate microglia through MCP‐1 release during cerebral hypoperfusion (i.e., hypoxia), leading to increases in SGLT1 gene expression in the neurons through IL‐1β released from microglia and macrophages.

We examined the effects of pre‐ and post‐treatment with mizagliflozin on behavioral performance of ACAS mice in wire hang, Y‐maze, and Morris water maze tests. The Morris water maze test demonstrated that the escape latency was significantly shorter in pre mizagliflozin‐treated ACAS mice than in sham‐operated mice, whereas it was longer in post mizagliflozin‐treated ACAS mice than in sham‐operated mice, suggesting that pre‐treatment with mizagliflozin is more effective to improve VCI in small vessel disease. Nevertheless, the wire hang and Y‐maze tests demonstrated that the latency to fall, spatial working memory, and spontaneous activity were similar among pre and post mizagliflozin‐treated ACAS and sham‐operated mice. Moreover, mizagliflozin ameliorated IL‐1β‐induced decreases in the survival rate of PC12HS cells even when these drugs began to be administered 6 h after IL‐1β stimulation started. These results suggest that post treatment with mizagliflozin is still effective to improve neural damage to some extent in small vessel disease.

Our previous study demonstrated that the latency to fall tested with the wire hang test was significantly shorter in sham‐operated SGLT1‐KO than sham‐operated WT mice, indicating that muscle strength, motivation not to fall, and/or CNS motor function were significantly impaired in SGLT‐KO mice regardless of ACAS. In contrast, mizagliflozin improved the shortened the latency to fall to the normal level in ACAS mice. Although SGLT1 exists in skeletal muscles,^37^ our results suggest that mizagliflozin might not inhibit SGLT1 in skeletal muscles.

We demonstrated that phlorizin reversed the decreases in cerebral blood flow in ACAS mice, and that the cerebral blood flow was similar between phlorizin‐treated ACAS mice and sham‐operated mice. Moreover, all of the factors tested were similar between phlorizin‐treated ACAS mice and sham‐operated mice, suggesting that phlorizin ameliorates neural injury and VCI through the improvement of cerebral blood flow. However, the mechanism of phlorizin‐induced improvement of cerebral blood flow and in ACAS mice is unknown. The present study demonstrated that phlorizin increased GLUT1 and 3 gene expressions in ACAS compared with sham‐operated mice. It is known that GLUT1 is enriched in central nervous system endothelial cells (Harik et al., 1990).^38^ Moreover, GLUT1 haploinsufficiency in mice was reported to decrease brain glucose uptake, reduce cerebral blood flow, and increase BBB permeability.^39^ Although phlorizin inhibits GLUT1, phlorizin‐induced increases in GLUT1 gene expression in brain endothelial cells may contribute to the improvement of cerebral blood flow in ACAS mice. In contrast to GLUT1 haploinsufficiency, GLUT3 haploinsufficiency did not affect glucose uptake into brains,^40^ suggesting that changes in GLUT3 expression do not affect brain function. Phlorizin did not reduce IL‐1β‐induced increases in SGLT1 and MCP‐1 gene expressions, whereas it ameliorated IL‐1β‐induced cell death in PC12HS cells. These results suggest that phlorizin improves hippocampal neuronal death, leading to VCI, through an inhibitory action against neuronal SGLT1 in a mouse model of ACAS‐induced vascular dementia. However, as phlorizin improved the increased IL‐1β gene expression in ACAS mice it may not affect hippocampal neuronal death, leading to VCI, through an inhibitory action against neuronal SGLT1 in a mouse model of ACAS‐induced vascular dementia.

This study has study limitations. SGLT1 existed in neurons and brain small vessel endothelial cells but not in glial cells.^23^, ^24^ However, recent studies showed that SGLT1 may be expressed in glial cells because SGLT1 mRNA was observed in rat astrocytes,^41^ and SGLT1‐related immunoreactivity was reported in glial cells of the rat ventromedial hypothalamic nucleus.^42^ Nevertheless, whether microglia or astrocytes are involved in the protective effects of mizagliflozin is still unclear because different SGLT1 expressions in various organs of mice and rats have been confirmed using quantitative RT‐PCR.^43^ Therefore, the effects of mizagliflozin on microglia and astrocytes would be examined in future project.

In conclusion, we first demonstrated the possibility of pharmacological therapy using an SGLT1 blocker for the prevention of VCI and SGLT1 blocker‐induced improvement of VCI through the inhibition of neural SGLT1 in a mouse model of small vessel disease.

## ETHICS APPROVAL STATEMENT

The study was performed in strict accordance with the recommendations set in the Guide for Care and Use of Laboratory Animals published by the US National Institutes of Health. The Animal Care Committee of Iwate Medical University approved this study. The Committee on the Ethics of Animal Experiments of Iwate Medical University approved the protocol (Permit Number: 28–04). Sodium pentobarbital and isoflurane anesthesia was used during all surgery and all efforts were made to minimize suffering.

## DISCLOSURE

No author has an actual or perceived conflict of interest with the contents of this article.

## AUTHORS CONTRIBUTIONS

Participated in research design: Masamichi Hirose, Eiichi Taira, Atsushi Sanbe.

Conducted experiments: Nanae Ishida, Sachiko Sato, Maki Saito, Yu Tezuka.

Performed data analysis: Nanae Ishida, Sachiko Sato, Maki Saito, Yu Tezuka, Masamichi Hirose.

Wrote or contributed to the writing of the manuscript: Nanae Ishida, Masamichi Hirose, Maki Saito.

## Data Availability

The data that support the findings of this study are available from the corresponding author upon reasonable request.
